# (3-{[2,6-Bis(1-methyl­eth­yl)phen­yl]imino-κ*N*}-1-phenyl­but-1-en-1-olato-κ*O*)­di­methyl­aluminium

**DOI:** 10.1107/S1600536812005880

**Published:** 2012-03-03

**Authors:** Haijun Hao, Baichun Zhu, Jianjun Yi

**Affiliations:** a School of Science, Beijing University of Chemical Technology, PO Box 144, Beisanhuandong Road 15, Chaoyang District, 100029 Beijing, People’s Republic of China; b Key Laboratory for Synthetic Resin, Petrochemical Research Institute, PetroChina Company Limited, Block E, Jingxinyuan A, No. 25 Beiwucun Road, Haidian District, Beijing 100195, People’s Republic of China

## Abstract

The mol­ecular structure of the title compound, [Al(CH_3_)_2_(C_22_H_26_NO)], displays a monomer with the Al^III^ atom in a distorted tetra­hedral environment defined by two methyl groups and the N and O atoms of the chelating ketiminate anion. The O—Al—N bite angle of the chelating ligand is 94.14 (9)°. The O—C—C—C—N backbone of the ligand is nearly coplanar (r.m.s. deviation = 0.029 Å) and the Al atom deviates significantly from the mean plane by 0.525 (3) Å. In the crystal, weak inter­molecular C—H⋯O inter­actions are observed.

## Related literature

For the structures of related aluminium complexes, see: Yu *et al.* (2002 ▶). For the structures of nickel, palladium, iron and zinc complexes with related bidentate β-ketoiminate ligands, see: He *et al.* (2003 ▶); Li *et al.* (2005 ▶); Benito-Garagorri *et al.* (2005 ▶); Granum *et al.* (2011 ▶). For a description of the Cambridge Structural Database, see: Allen (2002 ▶).
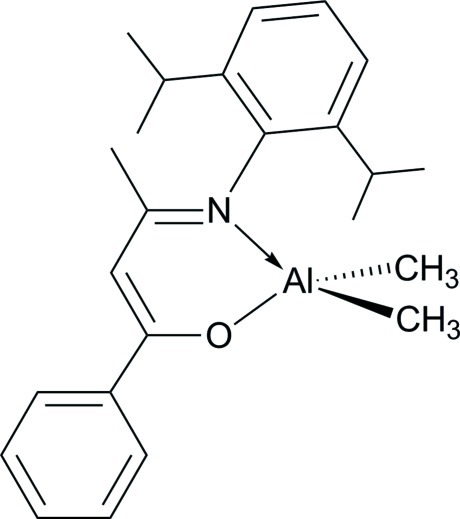


## Experimental

### 

#### Crystal data

[Al(CH_3_)_2_(C_22_H_26_NO)]
*M* *_r_* = 377.49
Monoclinic, 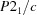

*a* = 15.231 (4) Å
*b* = 9.994 (3) Å
*c* = 15.289 (4) Å
β = 102.889 (5)°
*V* = 2268.7 (10) Å^3^
*Z* = 4
Mo *K*α radiation
μ = 0.10 mm^−1^
*T* = 293 K
0.20 × 0.18 × 0.12 mm

#### Data collection

Bruker SMART CCD area-detector diffractometer
Absorption correction: multi-scan *SADABS* (Bruker, 1998 ▶) *T*_min_ = 0.973, *T*_max_ = 0.988
12801 measured reflections
4662 independent reflections
2308 reflections with *I* > 2σ(*I*)
*R*_int_ = 0.071

#### Refinement

*R*[*F* ^2^ > 2σ(*F* ^2^)] = 0.062
*wR*(*F* ^2^) = 0.142
*S* = 0.99
4662 reflections
251 parameters
H-atom parameters constrained
Δρ_max_ = 0.18 e Å^−3^
Δρ_min_ = −0.18 e Å^−3^

### 

Data collection: *SMART* (Bruker, 1998 ▶); cell refinement: *SMART* and *SAINT* (Bruker, 1998 ▶); data reduction: *SAINT*; program(s) used to solve structure: *SHELXS97* (Sheldrick, 2008 ▶); program(s) used to refine structure: *SHELXL97* (Sheldrick, 2008 ▶); molecular graphics: *SHELXTL* (Sheldrick, 2008 ▶); software used to prepare material for publication: *SHELXTL*.

## Supplementary Material

Crystal structure: contains datablock(s) I, global. DOI: 10.1107/S1600536812005880/zq2142sup1.cif

Structure factors: contains datablock(s) I. DOI: 10.1107/S1600536812005880/zq2142Isup3.hkl

Supplementary material file. DOI: 10.1107/S1600536812005880/zq2142Isup4.cdx

Additional supplementary materials:  crystallographic information; 3D view; checkCIF report

## Figures and Tables

**Table 1:** Selected bond lengths (Å)

Al1—O1	1.7853 (19)
Al1—C24	1.939 (3)
Al1—N1	1.947 (2)
Al1—C23	1.949 (3)

**Table 2:** Hydrogen-bond geometry (Å, °)

*D*—H⋯*A*	*D*—H	H⋯*A*	*D*⋯*A*	*D*—H⋯*A*
C5—H5⋯O1^i^	0.93	2.69	3.525 (2)	151

Symmetry code: (i) 
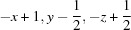
.

**Fig. 1.**
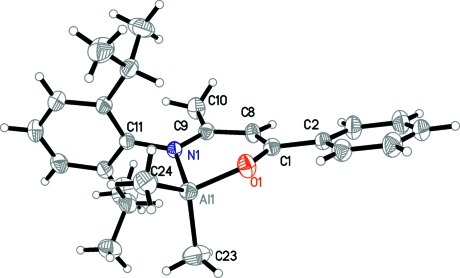
The molecular structure of the title compound with displacement ellipsoids drawn at the 30% probability level.

## supplementary crystallographic information

### Comment

The bidentate β-ketoiminate ligand, {[2,6-bis(1-methylethyl)phenyl]imino}pent-2-en-2-olate, is well known in the literature in transition-metal complexes (see Granum, *et al.*, 2011, and references therein) while the phenyl derivative used in the title compound, {[2,6-bis(1-methylethyl)phenyl]imino}-1-phenylbut-1-en-1-olate, is only reported in the Cambridge Structural Database (Allen, 2002) for two Ni complexes (He *et al.*, 2003; Li *et al.*, 2005) and one Pd complex (Benito-Garagorri *et al.*, 2005).

The molecular structure of the title compound, C_24_H_32_AlNO, displays a monomer with the Al atom in a distorted tetrahedral environment defined by two methyl groups and the N and O atoms of the chelating ketiminate ligand. (Yu, *et al.*, 2002). The Al—O1, Al1—N1 (Table 1), C1—C8, and C8—C9 bond lengths of 1.7853 (10), 1.947 (2), 1.360 (3) and1.417 (3) Å, respectively, are very similar to those in related aluminium compounds (Yu *et al.*, 2002). The biting angle of the ligand, O1—Al1—N1, is 94.14 (9)°. The backbone of the ligand, O1–C1–C8–C9–N1, is nearly coplanar (r.m.s. deviation = 0.029 Å) and the Al atom significantly deviates from the mean plane by 0.525 (3) Å. In the crystal structure, weak intermolecular C–H···O are observed (Table 2).

### Experimental

All the operations were carried out by using Schlenk techniques or in a drybox under dinitrogen atmosphere. The ketiminate ligand [PhC(O)CHCMeNHAr] (0.645 g, 2.00 mmol) was dissolved in 30 ml of toluene. To this solution, AlMe_3_ (1.1 ml, 2.0 *M* in toluene, 2.2 mmol) was added dropwise at room temperature and the resulting solution was stirred for 3 h. After the removal of all volatiles, the residue was dissolved in hexane and stored at 0 °C for 12 h to afford colorless crystals (0.67 g, 90%), which were suitable for a X-ray diffraction analysis.

### Refinement

All hydrogen positions were calculated after each cycle of refinement using a riding model, with C—H = 0.93Å and *U*_iso_(H) = 1.2*U*_eq_(C) for aromatic H atoms, with C—H = 0.98Å and *U*_iso_(H) = 1.2*U*_eq_(C) for methine H atoms, and with C—H = 0.96Å and *U*_iso_(H) = 1.5*U*_eq_(C) for methyl H atoms.

### Crystal data

**Table:**

[Al(CH_3_)_2_(C_22_H_26_NO)]	*F*(000) = 816
*M**_r_* = 377.49	*D*_x_ = 1.105 Mg m^−^^3^
Monoclinic, *P*2_1_/*c*	Mo *K*α radiation, λ = 0.71073 Å
Hall symbol: -P 2ybc	Cell parameters from 960 reflections
*a* = 15.231 (4) Å	θ = 2.5–22.1°
*b* = 9.994 (3) Å	µ = 0.10 mm^−^^1^
*c* = 15.289 (4) Å	*T* = 293 K
β = 102.889 (5)°	Colourless, colourless
*V* = 2268.7 (10) Å^3^	0.20 × 0.18 × 0.12 mm
*Z* = 4	

### Data collection

**Table:**

Bruker SMART CCD area-detector diffractometer	4662 independent reflections
Radiation source: fine-focus sealed tube	2308 reflections with *I* > 2σ(*I*)
Graphite monochromator	*R*_int_ = 0.071
phi and ω scans	θ_max_ = 26.5°, θ_min_ = 1.4°
Absorption correction: multi-scan *SADABS* (Bruker, 1998)	*h* = −19→15
*T*_min_ = 0.973, *T*_max_ = 0.988	*k* = −12→12
12801 measured reflections	*l* = −19→19

### Refinement

**Table:**

Refinement on *F*^2^	Primary atom site location: structure-invariant direct methods
Least-squares matrix: full	Secondary atom site location: difference Fourier map
*R*[*F*^2^ > 2σ(*F*^2^)] = 0.062	Hydrogen site location: inferred from neighbouring sites
*wR*(*F*^2^) = 0.142	H-atom parameters constrained
*S* = 0.99	*w* = 1/[σ^2^(*F*_o_^2^) + (0.0554*P*)^2^ + 0.1518*P*] where *P* = (*F*_o_^2^ + 2*F*_c_^2^)/3
4662 reflections	(Δ/σ)_max_ = 0.002
251 parameters	Δρ_max_ = 0.18 e Å^−^^3^
0 restraints	Δρ_min_ = −0.18 e Å^−^^3^

### Special details

**Table:**

Geometry. All e.s.d.'s (except the e.s.d. in the dihedral angle between two l.s. planes) are estimated using the full covariance matrix. The cell e.s.d.'s are taken into account individually in the estimation of e.s.d.'s in distances, angles and torsion angles; correlations between e.s.d.'s in cell parameters are only used when they are defined by crystal symmetry. An approximate (isotropic) treatment of cell e.s.d.'s is used for estimating e.s.d.'s involving l.s. planes.
Refinement. Refinement of *F*^2^ against ALL reflections. The weighted *R*-factor *wR* and goodness of fit *S* are based on *F*^2^, conventional *R*-factors *R* are based on *F*, with *F* set to zero for negative *F*^2^. The threshold expression of *F*^2^ > σ(*F*^2^) is used only for calculating *R*-factors(gt) *etc*. and is not relevant to the choice of reflections for refinement. *R*-factors based on *F*^2^ are statistically about twice as large as those based on *F*, and *R*- factors based on ALL data will be even larger.

### Fractional atomic coordinates and isotropic or equivalent isotropic displacement parameters (Å^2^)

**Table:**

	*x*	*y*	*z*	*U*_iso_*/*U*_eq_	
Al1	0.72813 (6)	0.07289 (8)	0.08417 (5)	0.0456 (3)	
O1	0.66287 (12)	−0.00347 (19)	0.15410 (12)	0.0557 (5)	
N1	0.79222 (13)	0.1912 (2)	0.17815 (13)	0.0380 (5)	
C1	0.68754 (17)	−0.0199 (3)	0.24069 (18)	0.0409 (7)	
C2	0.63259 (19)	−0.1183 (3)	0.27835 (19)	0.0431 (7)	
C3	0.6553 (2)	−0.1596 (3)	0.3665 (2)	0.0639 (9)	
H3	0.7068	−0.1255	0.4045	0.077*	
C4	0.6024 (3)	−0.2510 (3)	0.3991 (2)	0.0732 (10)	
H4	0.6184	−0.2784	0.4587	0.088*	
C5	0.5262 (2)	−0.3012 (3)	0.3435 (3)	0.0678 (10)	
H5	0.4901	−0.3618	0.3655	0.081*	
C6	0.5035 (2)	−0.2623 (3)	0.2560 (3)	0.0662 (9)	
H6	0.4522	−0.2971	0.2180	0.079*	
C7	0.5564 (2)	−0.1714 (3)	0.2236 (2)	0.0572 (8)	
H7	0.5404	−0.1456	0.1637	0.069*	
C8	0.75586 (18)	0.0519 (3)	0.29209 (17)	0.0456 (7)	
H8	0.7741	0.0273	0.3521	0.055*	
C9	0.80191 (17)	0.1606 (3)	0.26307 (18)	0.0415 (7)	
C10	0.8627 (2)	0.2406 (3)	0.33578 (18)	0.0608 (9)	
H10A	0.9177	0.1922	0.3576	0.091*	
H10B	0.8331	0.2554	0.3842	0.091*	
H10C	0.8760	0.3251	0.3119	0.091*	
C11	0.82742 (18)	0.3144 (3)	0.14972 (16)	0.0396 (7)	
C12	0.76949 (18)	0.4247 (3)	0.13277 (17)	0.0441 (7)	
C13	0.67952 (19)	0.4276 (3)	0.1588 (2)	0.0565 (8)	
H13	0.6605	0.3347	0.1635	0.068*	
C14	0.6892 (2)	0.4919 (4)	0.2512 (2)	0.0892 (12)	
H14A	0.7336	0.4442	0.2943	0.134*	
H14B	0.6324	0.4886	0.2685	0.134*	
H14C	0.7076	0.5835	0.2487	0.134*	
C15	0.6056 (2)	0.4978 (4)	0.0913 (2)	0.0863 (11)	
H15A	0.6172	0.5923	0.0928	0.129*	
H15B	0.5485	0.4815	0.1062	0.129*	
H15C	0.6043	0.4640	0.0322	0.129*	
C16	0.7996 (2)	0.5371 (3)	0.09505 (19)	0.0603 (9)	
H16	0.7624	0.6118	0.0831	0.072*	
C17	0.8826 (2)	0.5407 (3)	0.0750 (2)	0.0692 (10)	
H17	0.9008	0.6164	0.0483	0.083*	
C18	0.9385 (2)	0.4338 (3)	0.0939 (2)	0.0617 (9)	
H18	0.9953	0.4384	0.0809	0.074*	
C19	0.91372 (19)	0.3177 (3)	0.13219 (17)	0.0459 (7)	
C20	0.9802 (2)	0.2053 (3)	0.1555 (2)	0.0604 (9)	
H20	0.9510	0.1321	0.1808	0.072*	
C21	1.0103 (3)	0.1520 (4)	0.0738 (2)	0.0964 (13)	
H21A	0.9584	0.1289	0.0280	0.145*	
H21B	1.0470	0.0739	0.0903	0.145*	
H21C	1.0445	0.2194	0.0515	0.145*	
C22	1.0629 (2)	0.2489 (4)	0.2259 (3)	0.1135 (15)	
H22A	1.0966	0.3132	0.2001	0.170*	
H22B	1.1001	0.1724	0.2460	0.170*	
H22C	1.0441	0.2884	0.2760	0.170*	
C23	0.8091 (2)	−0.0611 (3)	0.0531 (2)	0.0842 (11)	
H23A	0.8551	−0.0821	0.1052	0.126*	
H23B	0.8364	−0.0265	0.0069	0.126*	
H23C	0.7756	−0.1405	0.0319	0.126*	
C24	0.6516 (2)	0.1673 (3)	−0.01497 (19)	0.0738 (10)	
H24A	0.6279	0.1052	−0.0622	0.111*	
H24B	0.6862	0.2346	−0.0369	0.111*	
H24C	0.6028	0.2090	0.0052	0.111*	

### Atomic displacement parameters (Å^2^)

**Table:**

	*U*^11^	*U*^22^	*U*^33^	*U*^12^	*U*^13^	*U*^23^
Al1	0.0502 (5)	0.0460 (5)	0.0408 (5)	−0.0029 (4)	0.0106 (4)	−0.0022 (4)
O1	0.0552 (13)	0.0653 (13)	0.0444 (12)	−0.0169 (10)	0.0061 (10)	0.0040 (10)
N1	0.0396 (13)	0.0369 (13)	0.0384 (13)	0.0003 (10)	0.0109 (10)	0.0035 (10)
C1	0.0380 (16)	0.0405 (16)	0.0437 (17)	0.0039 (13)	0.0079 (14)	0.0043 (13)
C2	0.0416 (17)	0.0389 (16)	0.0508 (18)	0.0000 (13)	0.0148 (15)	0.0014 (14)
C3	0.072 (2)	0.062 (2)	0.058 (2)	−0.0226 (18)	0.0151 (18)	0.0045 (17)
C4	0.092 (3)	0.067 (2)	0.068 (2)	−0.015 (2)	0.033 (2)	0.0093 (18)
C5	0.068 (2)	0.052 (2)	0.095 (3)	−0.0082 (18)	0.043 (2)	0.003 (2)
C6	0.046 (2)	0.061 (2)	0.091 (3)	−0.0111 (17)	0.0165 (19)	0.0031 (19)
C7	0.048 (2)	0.057 (2)	0.066 (2)	−0.0031 (16)	0.0128 (17)	0.0041 (16)
C8	0.0488 (18)	0.0498 (18)	0.0369 (16)	−0.0022 (15)	0.0067 (14)	0.0091 (13)
C9	0.0399 (17)	0.0406 (16)	0.0435 (18)	0.0003 (13)	0.0083 (13)	0.0027 (13)
C10	0.067 (2)	0.065 (2)	0.0466 (18)	−0.0177 (18)	0.0033 (16)	−0.0002 (15)
C11	0.0438 (18)	0.0382 (17)	0.0372 (16)	−0.0033 (13)	0.0099 (13)	−0.0001 (12)
C12	0.0498 (18)	0.0394 (16)	0.0441 (16)	−0.0032 (15)	0.0122 (14)	−0.0003 (14)
C13	0.054 (2)	0.0423 (17)	0.076 (2)	0.0048 (16)	0.0201 (17)	0.0100 (16)
C14	0.092 (3)	0.110 (3)	0.076 (2)	0.031 (2)	0.040 (2)	−0.001 (2)
C15	0.066 (2)	0.084 (3)	0.107 (3)	0.021 (2)	0.015 (2)	0.015 (2)
C16	0.075 (2)	0.043 (2)	0.065 (2)	0.0018 (16)	0.0207 (19)	0.0063 (15)
C17	0.084 (3)	0.055 (2)	0.077 (2)	−0.013 (2)	0.037 (2)	0.0131 (18)
C18	0.060 (2)	0.063 (2)	0.070 (2)	−0.0121 (19)	0.0315 (18)	0.0002 (18)
C19	0.0466 (18)	0.0487 (19)	0.0436 (17)	−0.0028 (15)	0.0129 (14)	0.0008 (14)
C20	0.0451 (19)	0.068 (2)	0.072 (2)	0.0035 (17)	0.0217 (17)	0.0097 (18)
C21	0.106 (3)	0.094 (3)	0.106 (3)	0.029 (3)	0.058 (3)	0.006 (2)
C22	0.060 (3)	0.150 (4)	0.116 (3)	0.001 (3)	−0.010 (2)	0.017 (3)
C23	0.080 (3)	0.075 (2)	0.100 (3)	0.004 (2)	0.025 (2)	−0.027 (2)
C24	0.092 (3)	0.076 (2)	0.048 (2)	0.006 (2)	0.0041 (18)	−0.0002 (17)

### Geometric parameters (Å, º)

**Table:**

Al1—O1	1.7853 (19)	C13—C14	1.528 (4)
Al1—C24	1.939 (3)	C13—H13	0.9800
Al1—N1	1.947 (2)	C14—H14A	0.9600
Al1—C23	1.949 (3)	C14—H14B	0.9600
O1—C1	1.303 (3)	C14—H14C	0.9600
N1—C9	1.310 (3)	C15—H15A	0.9600
N1—C11	1.447 (3)	C15—H15B	0.9600
C1—C8	1.360 (3)	C15—H15C	0.9600
C1—C2	1.488 (3)	C16—C17	1.367 (4)
C2—C7	1.377 (4)	C16—H16	0.9300
C2—C3	1.377 (4)	C17—C18	1.357 (4)
C3—C4	1.384 (4)	C17—H17	0.9300
C3—H3	0.9300	C18—C19	1.390 (4)
C4—C5	1.371 (4)	C18—H18	0.9300
C4—H4	0.9300	C19—C20	1.501 (4)
C5—C6	1.362 (4)	C20—C21	1.519 (4)
C5—H5	0.9300	C20—C22	1.527 (4)
C6—C7	1.377 (4)	C20—H20	0.9800
C6—H6	0.9300	C21—H21A	0.9600
C7—H7	0.9300	C21—H21B	0.9600
C8—C9	1.417 (3)	C21—H21C	0.9600
C8—H8	0.9300	C22—H22A	0.9600
C9—C10	1.508 (4)	C22—H22B	0.9600
C10—H10A	0.9600	C22—H22C	0.9600
C10—H10B	0.9600	C23—H23A	0.9600
C10—H10C	0.9600	C23—H23B	0.9600
C11—C19	1.399 (3)	C23—H23C	0.9600
C11—C12	1.400 (4)	C24—H24A	0.9600
C12—C16	1.386 (4)	C24—H24B	0.9600
C12—C13	1.510 (4)	C24—H24C	0.9600
C13—C15	1.519 (4)		
			
O1—Al1—C24	111.00 (13)	C13—C14—H14A	109.5
O1—Al1—N1	94.14 (9)	C13—C14—H14B	109.5
C24—Al1—N1	113.34 (12)	H14A—C14—H14B	109.5
O1—Al1—C23	108.60 (13)	C13—C14—H14C	109.5
C24—Al1—C23	116.53 (15)	H14A—C14—H14C	109.5
N1—Al1—C23	110.90 (13)	H14B—C14—H14C	109.5
C1—O1—Al1	126.10 (18)	C13—C15—H15A	109.5
C9—N1—C11	122.0 (2)	C13—C15—H15B	109.5
C9—N1—Al1	121.08 (18)	H15A—C15—H15B	109.5
C11—N1—Al1	116.95 (16)	C13—C15—H15C	109.5
O1—C1—C8	122.0 (2)	H15A—C15—H15C	109.5
O1—C1—C2	114.6 (2)	H15B—C15—H15C	109.5
C8—C1—C2	123.3 (2)	C17—C16—C12	121.5 (3)
C7—C2—C3	118.3 (3)	C17—C16—H16	119.2
C7—C2—C1	119.3 (3)	C12—C16—H16	119.2
C3—C2—C1	122.4 (3)	C18—C17—C16	120.0 (3)
C2—C3—C4	120.7 (3)	C18—C17—H17	120.0
C2—C3—H3	119.6	C16—C17—H17	120.0
C4—C3—H3	119.6	C17—C18—C19	122.1 (3)
C5—C4—C3	119.9 (3)	C17—C18—H18	119.0
C5—C4—H4	120.0	C19—C18—H18	119.0
C3—C4—H4	120.0	C18—C19—C11	117.1 (3)
C6—C5—C4	119.9 (3)	C18—C19—C20	119.5 (3)
C6—C5—H5	120.0	C11—C19—C20	123.4 (2)
C4—C5—H5	120.0	C19—C20—C21	112.3 (3)
C5—C6—C7	120.1 (3)	C19—C20—C22	111.1 (3)
C5—C6—H6	120.0	C21—C20—C22	109.1 (3)
C7—C6—H6	120.0	C19—C20—H20	108.1
C2—C7—C6	121.1 (3)	C21—C20—H20	108.1
C2—C7—H7	119.5	C22—C20—H20	108.1
C6—C7—H7	119.5	C20—C21—H21A	109.5
C1—C8—C9	126.3 (2)	C20—C21—H21B	109.5
C1—C8—H8	116.9	H21A—C21—H21B	109.5
C9—C8—H8	116.9	C20—C21—H21C	109.5
N1—C9—C8	122.4 (2)	H21A—C21—H21C	109.5
N1—C9—C10	121.4 (2)	H21B—C21—H21C	109.5
C8—C9—C10	116.2 (2)	C20—C22—H22A	109.5
C9—C10—H10A	109.5	C20—C22—H22B	109.5
C9—C10—H10B	109.5	H22A—C22—H22B	109.5
H10A—C10—H10B	109.5	C20—C22—H22C	109.5
C9—C10—H10C	109.5	H22A—C22—H22C	109.5
H10A—C10—H10C	109.5	H22B—C22—H22C	109.5
H10B—C10—H10C	109.5	Al1—C23—H23A	109.5
C19—C11—C12	121.8 (2)	Al1—C23—H23B	109.5
C19—C11—N1	120.3 (2)	H23A—C23—H23B	109.5
C12—C11—N1	117.7 (2)	Al1—C23—H23C	109.5
C16—C12—C11	117.5 (2)	H23A—C23—H23C	109.5
C16—C12—C13	119.9 (3)	H23B—C23—H23C	109.5
C11—C12—C13	122.5 (2)	Al1—C24—H24A	109.5
C12—C13—C15	114.1 (2)	Al1—C24—H24B	109.5
C12—C13—C14	110.2 (3)	H24A—C24—H24B	109.5
C15—C13—C14	109.7 (3)	Al1—C24—H24C	109.5
C12—C13—H13	107.5	H24A—C24—H24C	109.5
C15—C13—H13	107.5	H24B—C24—H24C	109.5
C14—C13—H13	107.5		
			
C24—Al1—O1—C1	−146.8 (2)	C1—C8—C9—N1	−10.8 (4)
N1—Al1—O1—C1	−29.8 (2)	C1—C8—C9—C10	168.7 (3)
C23—Al1—O1—C1	83.9 (2)	C9—N1—C11—C19	92.0 (3)
O1—Al1—N1—C9	24.7 (2)	Al1—N1—C11—C19	−88.7 (3)
C24—Al1—N1—C9	139.7 (2)	C9—N1—C11—C12	−93.7 (3)
C23—Al1—N1—C9	−87.0 (2)	Al1—N1—C11—C12	85.6 (2)
O1—Al1—N1—C11	−154.67 (18)	C19—C11—C12—C16	2.0 (4)
C24—Al1—N1—C11	−39.7 (2)	N1—C11—C12—C16	−172.2 (2)
C23—Al1—N1—C11	93.6 (2)	C19—C11—C12—C13	−174.4 (2)
Al1—O1—C1—C8	18.8 (4)	N1—C11—C12—C13	11.4 (4)
Al1—O1—C1—C2	−163.72 (17)	C16—C12—C13—C15	41.1 (4)
O1—C1—C2—C7	−7.2 (4)	C11—C12—C13—C15	−142.6 (3)
C8—C1—C2—C7	170.2 (3)	C16—C12—C13—C14	−82.9 (3)
O1—C1—C2—C3	172.3 (3)	C11—C12—C13—C14	93.4 (3)
C8—C1—C2—C3	−10.3 (4)	C11—C12—C16—C17	0.0 (4)
C7—C2—C3—C4	−0.6 (4)	C13—C12—C16—C17	176.5 (3)
C1—C2—C3—C4	179.8 (3)	C12—C16—C17—C18	−1.6 (5)
C2—C3—C4—C5	−0.2 (5)	C16—C17—C18—C19	1.2 (5)
C3—C4—C5—C6	0.9 (5)	C17—C18—C19—C11	0.7 (4)
C4—C5—C6—C7	−0.8 (5)	C17—C18—C19—C20	−177.2 (3)
C3—C2—C7—C6	0.8 (4)	C12—C11—C19—C18	−2.3 (4)
C1—C2—C7—C6	−179.7 (3)	N1—C11—C19—C18	171.7 (2)
C5—C6—C7—C2	−0.1 (5)	C12—C11—C19—C20	175.5 (3)
O1—C1—C8—C9	7.3 (4)	N1—C11—C19—C20	−10.5 (4)
C2—C1—C8—C9	−170.0 (2)	C18—C19—C20—C21	−60.2 (4)
C11—N1—C9—C8	169.6 (2)	C11—C19—C20—C21	122.0 (3)
Al1—N1—C9—C8	−9.7 (3)	C18—C19—C20—C22	62.2 (4)
C11—N1—C9—C10	−9.9 (4)	C11—C19—C20—C22	−115.5 (3)
Al1—N1—C9—C10	170.84 (19)		

### Hydrogen-bond geometry (Å, º)

**Table:**

*D*—H···*A*	*D*—H	H···*A*	*D*···*A*	*D*—H···*A*
C5—H5···O1^i^	0.93	2.69	3.525 (2)	151

Symmetry code: (i) −*x*+1, *y*−1/2, −*z*+1/2.
